# Population genomic analyses suggest recent dispersal events of the pathogen *Cercospora zeina* into East and Southern African maize cropping systems

**DOI:** 10.1093/g3journal/jkad214

**Published:** 2023-09-20

**Authors:** Tanya Welgemoed, Tuan A Duong, Irene Barnes, Eva H Stukenbrock, Dave K Berger

**Affiliations:** Department of Biochemistry, Genetics and Microbiology, Forestry and Agricultural Biotechnology Institute, University of Pretoria, Private Bag X20, Hatfield 0028, South Africa; Department of Biochemistry, Genetics and Microbiology, Forestry and Agricultural Biotechnology Institute, University of Pretoria, Private Bag X20, Hatfield 0028, South Africa; Department of Biochemistry, Genetics and Microbiology, Forestry and Agricultural Biotechnology Institute, University of Pretoria, Private Bag X20, Hatfield 0028, South Africa; Environmental Genomics, Christian-Albrechts University of Kiel, Am Botanischen Garten 1-11, Kiel 24118, Germany; Max Planck Institute for Evolutionary Biology, August-Thienemann-Str. 2, Plön 24306, Germany; Department of Plant and Soil Sciences, Forestry and Agricultural Biotechnology Institute, University of Pretoria, Private Bag X20, Hatfield 0028, South Africa

**Keywords:** crop pathogen, pangenome, gray leaf spot, population genomics, *Cercospora*, maize, corn

## Abstract

A serious factor hampering global maize production is gray leaf spot disease. *Cercospora zeina* is one of the causative pathogens, but population genomics analysis of *C. zeina* is lacking. We conducted whole-genome Illumina sequencing of a representative set of 30 *C. zeina* isolates from Kenya and Uganda (East Africa) and Zambia, Zimbabwe, and South Africa (Southern Africa). Selection of the diverse set was based on microsatellite data from a larger collection of the pathogen. Pangenome analysis of the *C. zeina* isolates was done by (1) de novo assembly of the reads with SPAdes, (2) annotation with BRAKER, and (3) protein clustering with OrthoFinder. A published long-read assembly of *C. zeina* (CMW25467) from Zambia was included and annotated using the same pipeline. This analysis revealed 790 non-shared accessory and 10,677 shared core orthogroups (genes) between the 31 isolates. Accessory gene content was largely shared between isolates from all countries, with a few genes unique to populations from Southern Africa (32) or East Africa (6). There was a significantly higher proportion of effector genes in the accessory secretome (44%) compared to the core secretome (24%). PCA, ADMIXTURE, and phylogenetic analysis using a neighbor-net network indicated a population structure with a geographical subdivision between the East African isolates and the Southern African isolates, although gene flow was also evident. The small pangenome and partial population differentiation indicated recent dispersal of *C. zeina* into Africa, possibly from 2 regional founder populations, followed by recurrent gene flow owing to widespread maize production across sub-Saharan Africa.

## Introduction

The development of agriculture has shifted the co-evolution of plants and pathogens. In large parts of the world, diverse environments containing a variety of plant species have been replaced with dense, near identical monocultures in similar, artificially maintained environments across different continents (McDonald and Stukenbrock 2016; Corredor-Moreno and Saunders 2020). This has favored crop pathogens to evolve more rapidly and with more virulence than in wild systems, and each time host resistance is overcome, they more easily spread through the largely homogeneous and interconnected global agricultural systems (McDonald and Stukenbrock 2016; Möller and Stukenbrock 2017; Corredor-Moreno and Saunders 2020). This makes pathogen evolution an increasingly important consideration for crop disease management.

Gray leaf spot (GLS) is considered as one of the top 3 maize foliar fungal diseases globally, however, it has remained an understudied plant-pathosystem (Ward *et al.* 1999; Savary *et al.* 2019). GLS was first identified in the USA as a maize fungal disease where infection by the Dothideomycete fungus *Cercospora zeae-maydis* causes rectangular tan lesions in the maize leaves (Tehon and Daniels 1925). Through AFLP profiles and ITS sequencing, it was determined that *C. zeae-maydis* represents a species complex: *C. zeae-maydis* groups I and II (Wang *et al.* 1998). These groups were confirmed to be sister species using a multigene phylogeny, and subsequently, *C. zeae-maydis* group II was described as a distinct species, renamed as *Cercospora zeina* (Dunkle and Levy 2000; Crous *et al.* 2006). Both *C. zeina* and *C. zeae-maydis* are present in the USA (Wang *et al.* 1998), Brazil (Brunelli *et al.* 2008), and China (Liu and Xu 2013), but in African countries, only *C. zeina* has been identified as a causal pathogen (Meisel *et al.* 2009; Nsibo *et al.* 2021). Having first been reported in Africa in 1988 from South Africa (Ward *et al*. 1999), GLS has since been reported in Cameroon, DR Congo, Kenya, Uganda, Zambia, and Zimbabwe (Ward *et al.* 1999; Dunkle and Levy 2000).

Despite the global impact of GLS, little has been resolved about the evolutionary history of *C. zeina*. Early population genetic studies found highly similar AFLP results between *C. zeina* isolates from African countries and from the USA (Dunkle and Levy 2000). Similarly, AFLP analysis of *C. zeina* isolates from Africa, the USA, and Brazil also found very low levels of genetic diversity within and between those regions (Brunelli *et al.* 2008). Low genetic diversity is expected after a genetic bottleneck, which would be the case if the population on a new continent was founded by a limited number of invasive isolates. This lack of differentiation between *C. zeina* genotypes on separate continents suggested that they shared a recent origin (Dunkle and Levy 2000), but it is unresolved whether the ancestral population was from the African continent or the American continent. In contrast, a large-scale microsatellite analysis with better resolution of genetic variation among 964 African isolates of *C. zeina* found that the African population, overall, had high levels of genetic diversity and was structured in distinct geographical clusters (Nsibo *et al.* 2021). However, no significant difference between the level of genetic diversity in each African cluster was found that could indicate an ancestral population (Nsibo *et al.* 2021). Alternatively to a bottleneck in invasive isolates, there could have been an earlier bottleneck in the ancestral population of *C. zeina* if its progenitor made a single shift to the maize host, as has occurred with the shifting of *Magnaporthe oryzae* to the rice host from Setaria millet (Couch *et al*. 2005).

The population structure found between isolates of *C. zeina* from different regions could be further investigated using whole-genome comparisons and evaluating gene presence–absence variation. The first whole-genome sequence for *C. zeina* was determined from an African isolate CMW25467 using Illumina technology (Wingfield *et al.* 2017), and improved upon with a PacBio assembly (Wingfield *et al.* 2022). The genome of a *C. zeina* isolate from China is also now available and was determined with a nanopore assembly (Cheng *et al.* 2023).

The presence–absence variation of chromosomal fragments and genes represents an important component of the genomic diversity of many filamentous plant pathogens (Badet and Croll 2020). In fungal species exhibiting such presence–absence variation, each individual genome contains “core” genes, shared by all members of the species, and “accessory” genes, which are variable among genotypes (McCarthy and Fitzpatrick 2019). The complete genome content of the species, defined as the “pangenome”, then consists of the core genes and the set of accessory genes present among the individual isolates in the pangenome (McCarthy and Fitzpatrick 2019). Assembling pathogen pangenomes to capture this component of the genomic diversity can therefore give informative insight into the evolutionary potential of a species.

Several methods have been developed to capture the pangenome variation of fungal pathogen species: either by alignment of de novo genome assemblies or by mapping reads to a reference genome followed by de novo assembly of unmapped reads to build a pangenome (Potgieter *et al.* 2020; Tettelin and Medini 2020). While more computationally expensive, the former approach using individual de novo assemblies has the advantage of resulting in more complete sequences, especially in genomes which contain many repetitive elements, as is the case in *C. zeina* (Wingfield *et al.* 2022).

The functions of the accessory component in a pathogen are shaped by its particular evolutionary history (Badet and Croll 2020). This is often host adaptation, but could also be environmental adaptation. There are many examples of accessory regions contributing to pathogenicity of fungal plant pathogens and being enriched for effectors (Möller and Stukenbrock 2017; Bertazzoni *et al.* 2018). Examples in the Dothideomycetes are the T-toxin biosynthesis cluster in the maize pathogen *Cochliobolus heterostrophus* (Condon *et al.* 2013), and the AvrLM11 effector of the Brassica pathogen *Leptosphaeria maculans* (Balesdent *et al.* 2013). The pangenome of *Pyrenophora tritici-repentis* had a greater proportion of accessory genes in the nonpathogenic isolates than in the pathogenic isolates (Gourlie *et al.* 2022). The relative size of the accessory component in a pathogen is associated with increases in overall genetic diversity, e.g. sexual recombination, or decreases, e.g. population bottlenecks (Badet and Croll 2020). In the higher range, the percentage of accessory genes in the pangenome of the wheat pathogens, *P. tritici-repentis* and *Zymoseptoria tritici*, was 56% (Gourlie *et al.* 2022) and 42% (Plissonneau *et al.* 2018), respectively. On the lower range of accessory genes, the percentage of accessory genes ranged from 10 to 20% in the model fungal species, *Saccharomyces cerevisiae*, *Candida albicans*, and *Cryptococcus neoformans* (McCarthy and Fitzpatrick 2019). Exceptionally low proportions were found in the nonagricultural pathogens *Microbotryum lychnidis-dioicae* (2% accessory) and *Microbotryum silenes-dioicae* (0.5% accessory) (Hartmann *et al.* 2018).

There is currently a lack of population genomics analysis of *C. zeina* at the whole-genome level. Whole-genome data will facilitate informative genome comparisons in terms of polymorphisms and gene content variation within *C. zeina* subpopulations in Africa. Conducting a pangenome study of *C. zeina* will produce a useful resource by identifying and functionally annotating the currently unknown accessory genes. As reviewed above, functions related to *C. zeina*'s adaptation, such as effectors, are expected to be enriched in the accessory genes and can be captured by this resource. This study will focus on the *C. zeina* population in Africa. The GLS disease burden is especially relevant in Africa, as the maize host is a staple food crop and measured yield losses can be up to 65% (Ward *et al.* 1999). Previous resources created with the African population, namely the comprehensive isolate collection and microsatellite data (Nsibo *et al.* 2021) and access to a high-quality *C. zeina* reference genome assembled with long-read data (Wingfield *et al.* 2022), can now be built on for a population genetics comparison using whole-genome data. In this study, we set out to characterize variation in genome content and SNP-based population structure among isolates of *C. zeina* isolated from maize fields in the East and Southern African geographical regions.

## Materials and methods

### Selection of *C. zeina* isolates

A subset of 30 diverse *C. zeina* isolates were selected from a total of 1,002 isolates previously isolated from 5 African countries (South Africa, Zimbabwe, Zambia, Uganda, and Kenya) (Muller *et al.* 2016; Nsibo *et al.* 2021). To facilitate the selection, the R package, poppr (Kamvar *et al.* 2014), was used to estimate the pairwise genetic distance between the isolates based on Bruvo's distance (Bruvo *et al.* 2004) using previously generated microsatellite data (Supplementary Table 2 of Nsibo *et al.* (2021)). The pair of isolates with the largest pairwise genetic distance was used as a starting point for the selection. The isolate with the greatest distance to the previously selected isolates was found by taking the pairwise distances between a potential addition and each of the previous selected isolates, and then using a numpy array (van der Walt *et al.* 2011) to find the isolate where the minimum of the pairwise distances was greater than the minimum of other potential isolates. This would also ensure that no clones were selected. The numpy loop was repeated until 30 isolates were selected across the geographical regions.

To ensure all geographical regions were represented, a maximum of 6 isolates were considered for each country of origin, namely Uganda, Kenya, Zambia, South Africa, and Zimbabwe. The exception was Zambia, as the Zambian isolates consisted of a single phylogenetic cluster with a greater proportion of clones (Nsibo *et al.* 2021). Therefore, only 3 Zambian isolates were selected, and the isolate set was complemented with an additional 2 South African isolates and 1 Kenyan isolate (the countries which provided the most migrants in Nsibo *et al.* (2021)). There were no clones (based on microsatellite data) in the final set of *C. zeina* isolates. A “reference genome”, determined previously with PacBio sequencing (Wingfield *et al.* 2022), for the *C. zeina* isolate CMW25467 (Meisel *et al.* 2009) from Zambia, was also included in this study bringing the total number of isolates to 31. The *C. zeina* PacBio reference genome (NCBI Accession # MVDW02) consisted of 17 nuclear contigs, which were used in this study, and 5 small contigs of nonprotein coding and mitochondrial sequences, which were excluded from further analyses.

### Genome sequencing, assembly, and annotation

All *C. zeina* isolates from Nsibo *et al.* (2021) were maintained as glycerol stocks. The glycerol stocks of the selected isolates were revitalized on V8 media and maintained until sufficient biomass was obtained for DNA extractions. DNA was extracted from freeze-dried mycelium using the protocol described in Duong *et al.* (2013) and quantified with a Qubit 4 fluorometer. DNA libraries were constructed and 150 bp paired-end Illumina sequencing was done by the Max Planck-Genome-centre (Cologne, Germany). The sequenced reads were assessed for quality using FastQC (RRID:SCR_014583) (Andrews 2019). Low quality bases below a QV of 20 were masked instead of trimmed as a quality control measure (Yun and Yun 2014). No singleton reads were used.

Individual de novo assemblies were made from the selected 30 *C. zeina* isolates using SPAdes (RRID:SCR_000131) (Prjibelski *et al.* 2020). SPAdes was set to use the default k-mer sizes and to run its optional MismatchCorrector module. The quality and completeness of each genome assembly was assessed with QUAST (RRID:SCR_001228) (v5.0.2) (Gurevich *et al.* 2013) and BUSCO using the *Dothideomycetes* lineage dataset (RRID:SCR_015008) (v5.4.3) (Simão *et al.* 2015), respectively.

The 30 de novo assembled genomes and the *C. zeina* CMW25467 reference genome were, for the sake of consistency, annotated using the same BRAKER (RRID:SCR_018964) (v2.1.6) pipeline (Hoff *et al.* 2019; Brůna *et al.* 2021). The pipeline used SAMTOOLS (Li *et al.* 2009), BAMTOOLS (Barnett *et al.* 2011), and GENEMARK (Ter-Hovhannisyan *et al.* 2008). The parameters for AUGUSTUS (Stanke *et al.* 2006) were trained using RNA alignments as extrinsic evidence (Stanke *et al.* 2008; Lomsadze *et al.* 2014). The RNA-seq data (Swart *et al.* 2017) was obtained from in planta samples of field-grown maize infected with *C. zeina* (GSE94442) and in vitro samples of CMW25467 *C. zeina* cultures grown on various media (GSE90705).

### Gene content comparison

The gene content of the 31 *C. zeina* isolates were compared by using Orthofinder (Emms and Kelly 2015) to group the predicted proteins from each isolate into orthologous groups of genes (orthogroups). For genes which had more than 1 transcript predicted, the protein from the longest transcript was used. Orthogroups which were present in all 31 isolates were classified as containing the core sequences, and orthogroups which were absent from 1 to 29 genomes were classified as containing the accessory sequences.

The sets of isolates sharing present/absent orthogroups were visualized as an UpSet plot (Lex *et al.* 2014) using the R package, UpSetR (Conway *et al.* 2017). The pan- and core-genome accumulation curves were calculated using the R script from Méric *et al.* (2014). A custom GNU Awk (RRID:SCR_018211) script was used to convert the orthogroup table produced by ORTHOFINDER into a presence/absence matrix to use as input for the R scripts. The median values of the accumulation curve iterations were fitted to a power law regression in R to determine whether the pangenome was open or closed (Tettelin *et al.* 2008).

The orthogroups were annotated with protein domains and GO terms using InterProScan v5.29 (RRID:SCR_005829) (Jones *et al.* 2014). One protein sequence from each orthogroup was selected. Putative protein identities and functions of each orthogroup representative was determined by BLASTP with Diamond (Buchfink *et al.* 2015) against the NCBI nr database, which also included *C. zeina* CMW25467 proteins (NCBI Assembly MVDW01; Wingfield *et al.* (2017)).

GO terms enriched in the accessory orthogroups compared to the core orthogroups were determined using topGO (RRID:SCR_014798) (Alexa and Rahnenfuhrer 2021). The weight01 algorithm was used to make the GO graph, and the Fisher test was used to test for significance.

The InterProScan THHM and signal-P databases also allowed the identification of secreted proteins, defined as having been annotated with a eukaryotic signal-P domain, but without a transmembrane domain. Effectors present in the orthogroups of secreted proteins were predicted with EFFECTOR-P (Sperschneider and Dodds 2022). The proportion of the effectors in the core orthogroups and the proportion in the accessory orthogroups were compared in R with a 2-sample test for equality of proportions with continuity correction.

The distance between the predicted genes and the nearest transposable elements (TEs) annotated in the *C. zeina* CMW25467 reference genome (Wingfield *et al.* 2022) were calculated with the “closest” tool in BEDTools (RRID:SCR_006646) (Quinlan and Hall 2010). Using R, the median distance of the core genes to the nearest TE and the median distance of the accessory genes to the nearest TE were calculated, and the significance of the difference between the core and accessory was calculated using a 2-tailed Wilcoxon rank sum test.

### SNP calling

Paired alignments of the Illumina reads from each *C. zeina* isolate were made against the *C. zeina* CMW25467 PacBio reference genome with BWA MEM (Li and Durbin 2009). SNPs and indels were called using GATK (RRID:SCR_001876) HaplotypeCaller. This was done by implementing the GVCF workflow, and the parameters were set to use a ploidy of 1.

Due to the lack of pre-existing *C. zeina* SNP data, SNP calls were hard-filtered using GATK SelectVariants to remove low quality SNPs of SOR > 3, MQ < 45, DP < 10, and DP > 2006. The distributions of these metrics were visualized to assess the filtering. The indels were likewise filtered to remove sites of QUAL < 30, FS > 100, and SOR > 6. The SNPs were further filtered to retain only the biallelic sites without missing data. This filtered SNP dataset was used for the Discriminant Analysis of Principal Components (DAPC) analysis (see below).

The SNP dataset was further filtered for the PCA with Eigensoft, ADMIXTURE, and phylogenetic analyses (see below) to remove SNPs that were present in only 1 isolate, by applying a minimum allele frequency of 0.067 and a maximum allele frequency of 0.967. This was done to avoid over-fitting (Linck and Battey 2019).

For downstream analyses affected by linkage disequilibrium (PCA and ADMIXTURE analyses; see below), the SNP dataset was also LD pruned with PLINK (RRID:SCR_001757) (Chang *et al.* 2015) in 50 SNP variant windows with a sliding window of 10 variants, to remove SNPs above 0.1 correlation.

### Population structure analyses

The relationships between the *C. zeina* isolates based on variation in the biallelic SNPs were visualized using DAPC (Jombart *et al.* 2010) with the ADEGENET (Jombart 2008) package in R (RRID:SCR_001905). The positions and effect of the SNPs were determined using SnpEff (Cingolani *et al.* 2012). The threshold for selecting SNPs that had a major effect on the observed DAPC clusters was taken as the 0.995 quantile on the DAPC loading plot.

An additional PCA analysis was done with the methods and utilities provided in the Eigensoft (RRID:SCR_004965) software package (v 8.0.0) (Price *et al.* 2006). The LD-pruned SNP files from PLINK were converted into the EIGENSTRAT format using the Eigensoft convertf utility. The Eigensoft smartpca perl wrapper was used to run the principal component analysis and then apply the EIGENSTRAT stratification correction method to the PCA plot. The corrected PCA plot was plotted using the Eigensoft ploteig utility, and was labeled to show the countries of origins.

ADMIXTURE (RRID:SCR_001263) (Alexander *et al.* 2009) was used to analyze the LD-pruned SNPs for admixture between the 31 *C. zeina* isolates and subpopulations between them. Convergence between runs of the admixture analysis were visualized with PONG (Behr *et al.* 2016).

To determine the phylogenetic relationships between the *C. zeina* isolates, biallelic SNPs that were present in 2 or more isolates were imported into R using vcfR (Knaus and Grünwald 2017). The R package, POPPR (Kamvar *et al.* 2014), was used to construct a bitwise distance matrix from the SNPs, which was then used to produce a neighbor-joining tree with 1,000 bootstraps. The R package PHANGORN (Schliep *et al.* 2017) was used to create a neighbor net (Bryant 2003) from the distance matrix and to add confidence values to the neighbor net from the bootstrap tree. The network was visualized using SPLITSTREE v4 (Huson and Bryant 2006).

## Results

### Sequencing and assembly of African *C. zeina* isolates

A subset of 30 *C. zeina* isolates representing a broad spread of genetically distant genotypes from the 5 African countries in Eastern and Southern Africa were selected for Illumina sequencing (Supplementary Fig. 1) (Table 1). This was made up of 8 isolates from South Africa, 7 isolates from Kenya, 6 isolates from Uganda, 6 isolates from Zimbabwe, and 3 isolates from Zambia (Table 1). *C. zeina* is a heterothallic fungus (Muller *et al.* 2016), and the diversity of mating type alleles was previously assessed for these fungal populations. We selected representative isolates of both mating types, including 13 *MAT1-1* and 17 *MAT1-2* isolates (Table 1). The number of filtered Illumina read pairs obtained for each isolate ranged from 3,653,040 to 9,385,376 (Table 1).

**Table 1.** Illumina sequencing and reference genome mapping of the 30 African isolates of *C. zeina*.

Isolate	Country	District	Mating type*^a^*	Read pairs	Read pairs mapped to reference genome (%)*^b^*	Average read depth	Reference genome covered by reads (bp)*^c^* (%)
KE.KAK.344	Kenya	Kakamega	MAT1-1	9,235,123	89	41.9	41,249,360 (99%)
KE.KER.468	Kenya	Kericho	MAT1-1	7,091,540	90	31.0	41,210,329 (99%)
KE.KER.511	Kenya	Kericho	MAT1-1	7,984,292	90	35.6	41,204,784 (99%)
KE.KIS.232	Kenya	Kisumu	MAT1-1	8,068,594	90	36.3	41,300,563 (99%)
KE.KIT.758	Kenya	Kitale	MAT1-1	5,287,131	89	22.7	41,148,040 (99%)
KE.SIA.288	Kenya	Siaya	MAT1-1	4,989,094	90	22.3	41,071,763 (98%)
KE.TRS.520	Kenya	Trans-Nzoia	MAT1-1	4,793,708	88	20.8	41,138,073 (99%)
UG.FTP.009	Uganda	Fortportal	MAT1-2	8,234,724	90	37.4	41,283,179 (99%)
UG.GYZ.032	Uganda	Wakiso	MAT1-2	7,927,199	89	33.8	41,247,497 (99%)
UG.KPC.038	Uganda	Kapchorwa	MAT1-1	8,025,442	89	33.5	41,215,743 (99%)
UG.LIR.101	Uganda	Lira	MAT1-2	9,385,376	89	42.2	41,278,896 (99%)
UG.MSK.001	Uganda	Masaka	MAT1-2	6,906,197	89	31.1	41,159,959 (99%)
UG.NML.092	Uganda	Wakiso	MAT1-1	8,233,233	89	37.5	41,234,210 (99%)
ZA.BZN.009	South Africa	Mbizana	MAT1-2	6,179,751	89	27.0	41,227,006 (99%)
ZA.BZN.007	South Africa	Mbizana	MAT1-2	6,614,752	89	28.4	41,467,018 (99%)
ZA.CED.V02.124	South Africa	Cedara	MAT1-1	8,719,944	88	37.8	41,378,025 (99%)
ZA.CED.V05.074	South Africa	Cedara	MAT1-1	8,055,000	88	35.6	41,395,899 (99%)
ZA.CRG.097	South Africa	Hlanganani	MAT1-2	8,142,980	89	37.2	41,271,764 (99%)
ZA.EST.017	South Africa	Ntabamhlophe	MAT1-2	3,653,040	88	15.5	41,132,557 (99%)
ZA.NTB.069	South Africa	Ntabankulu	MAT1-1	8,035,504	88	33.6	41,259,132 (99%)
ZA.NXM.079	South Africa	KwaNxamalala	MAT1-1	6,127,175	91	26.9	41,265,685 (99%)
ZM.CHS.019	Zambia	Chisamba	MAT1-2	8,019,453	88	35.4	41,332,640 (99%)
ZM.CHS.045	Zambia	Chisamba	MAT1-1	6,044,013	89	26.6	41,280,858 (99%)
ZM.CHS.092	Zambia	Chisamba	MAT1-2	5,704,227	92	26.4	41,235,589 (99%)
ZW.AFR.270	Zimbabwe	Mutare	MAT1-1	6,512,663	91	28.8	41,265,177 (99%)
ZW.ART.151	Zimbabwe	Harare	MAT1-2	6,006,878	85	24.3	41,299,810 (99%)
ZW.CHN.315	Zimbabwe	Chinhoyi	MAT1-2	8,216,363	90	37.7	41,346,000 (99%)
ZW.CMH.326	Zimbabwe	Harare	MAT1-2	8,264,299	89	37.3	41,362,442 (99%)
ZW.RRS.263	Zimbabwe	KweKwe	MAT1-1	8,233,381	89	37.0	41,354,564 (99%)
ZW.STP.133	Zimbabwe	Harare	MAT1-1	8,221,698	90	37.8	41,366,302 (99%)

The mating type of each *C. zeina* isolate was confirmed based on the whole-genome data. The mating type of *C. zeina* CMW25467 is MAT1-1.

The percentage of read pairs from each isolate that was aligned against the *C. zeina* CMW25467 PacBio reference genome.

The total amount of base pairs and percentage of the *C. zeina* CMW25467 PacBio reference genome that was covered by read pairs from each isolate. The size of the reference genome in this study was 41,717,156 bp.

We hypothesized that geographic separation between the Eastern and Southern African regions or countries may have resulted in local adaptation possibly reflected in distinct gene contents. To this end, we addressed the composition of accessory genes in the *C. zeina* populations and conducted de novo genome assemblies to obtain the gene content in each isolate. Summary statistics of the de novo assemblies are listed in Supplementary Table 1. In brief, the de novo assemblies of the 30 genomes of *C. zeina* resulted in genome sizes of approximately 30 Mb, ranging from 29.2 Mb to 29.9 Mb suggesting a conserved genome composition among the isolates (Supplementary Table 1). N50 values of the de novo assemblies ranged from 74,006 to 90,244 bp (Supplementary Table 1), and the assemblies were similar in size to the non-repetitive component which made up approximately 2/3 of the 42 Mb *C. zeina* CMW25467 reference genome generated with PacBio sequencing (Wingfield *et al.* 2022). As expected, the Illumina sequencing could not resolve the repetitive components of the genome as fully as in the reference genome assembly generated by long-read sequencing (Wingfield *et al*. 2022). BUSCO annotations however indicated that the short-read genome assemblies had captured most of the protein-coding gene content since 97% of the *Dothideomycete* conserved gene set was present in each isolate (Supplementary Table 1).

### Gene content comparison of African *C. zeina* isolates

The BRAKER annotation pipeline predicted 11,071 protein-coding genes from the reference genome (Supplementary Table 1) and was thus more conservative than the annotations made in the previous study where 11,570 genes were predicted (Wingfield *et al.* 2022). The number of genes annotated in the 30 *C. zeina* isolates ranged from 11,135–11,194 (Supplementary Table 1), which were more than were predicted in the previous Illumina version of the reference genome (10,193) (Wingfield *et al.* 2017).

Core and accessory genes in *C. zeina* were identified in the 31 isolates by comparison of orthogroups (listed in Supplementary Table 2). First, genes were clustered into (1) 10,677 core orthogroups (93% of the orthogroups), of which 10,306 had a BLAST hit and 5,749 could be annotated with a GO term, and (2) 790 accessory orthogroups (7% of the orthogroups), of which 433 and 401 had a BLAST hit and GO term, respectively (Supplementary Table 2). Based on these analyses, we found that the number of accessory orthogroups in each isolate ranged from 394 to 517 (Supplementary Tables 1 and 2).

**Fig. 1.**
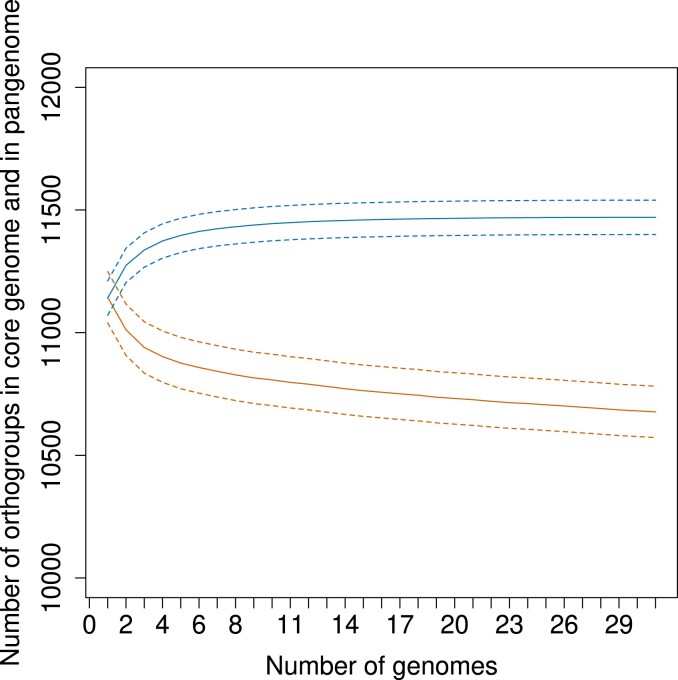
The *C. zeina* pangenome from the 31 African isolates shows a plateau in gene content. The graph illustrates the number of genes in the total pangenome (top blue line; accumulation curve) and the shared core genome (bottom orange line; rarefaction curve) counted by including genomes from additional *C. zeina* isolates. The solid lines represent the average gene number over 100 iterations, and the dotted lines represent the standard deviations.

Since more than 700 genes can be considered accessory, we further investigated the pangenome structure of *C. zeina*. The first step was to determine if we had sequenced sufficient *C. zeina* isolates to capture the accessory gene content. The exponent of the *C. zeina* pangenome accumulation curve was very close to 1 (0.99) (Fig. 1), indicating a very small accumulation of new genes with the successive inclusion of genomes from additional isolates. We therefore concluded that with 31 isolates, we had captured a representative pangenome of *C. zeina* isolates from the major maize producing countries in the Eastern and Southern African geographical regions.

**Fig. 2.**
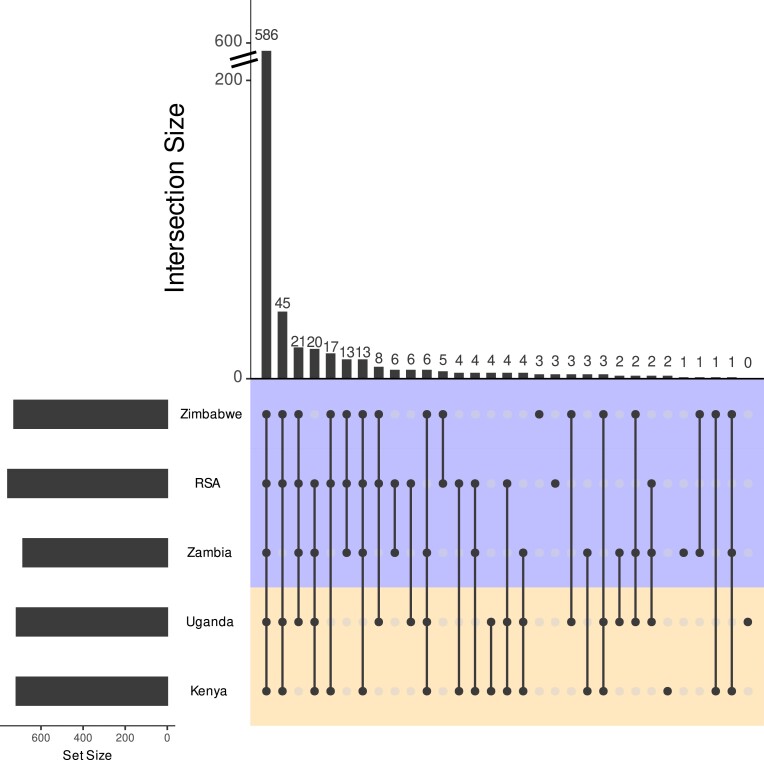
The variation in accessory gene content between the 31 *C. zeina* isolates does not follow a region-specific pattern. The UpSet plot shows the shared orthogroups between the isolates, with the intersection rows shaded based on country.

We had hypothesized that there may be regional (East vs Southern Africa) or country-specific accessory genes on account of local adaptation. We evaluated the distribution of the orthogroups between the regions and countries to determine whether there were any orthogroups specific to a region or country. The overlaps of the 790 accessory orthogroups between each country were visualized using an UpSet plot (Fig. 2). The majority of the accessory orthogroups (586 out of 790) were present in all 5 countries, and the rest of the orthogroups were randomly distributed between the countries and regions (Fig. 2). There were 32 orthogroups in total found only in at least one of the Southern African countries (Fig. 2). Of these, we could assign a GO term for 2; protein binding and sphingolipid metabolism (Table 2). Three of the 6 orthogroups found in only at least 1 East African country had GO terms (oxidoreductase activity, mitochondrial associated amino acid catabolism, protein binding) (Table 2). Overall, out of the orthogroups with GO terms that were common between the core and accessory, there were several GO terms related to transcription and kinase activity that were significantly enriched in the accessory genes compared to the core genes (*P* < 0.05) (Table 3).

**Table 2.** The GO terms (molecular function) of region-specific *C. zeina* accessory genes (orthogroups).

Region	Region-specific orthogroups	GO ID	GO annotation
Southern Africa	OG0011401	GO:0005515	Protein binding
	OG0011461	GO:0004348, GO:0006665	Glucosylceramidase activity; sphingolipid metabolic process
East Africa	OG0011402	GO:0005506; GO:0016705; GO:0020037; GO:0033566; GO:0004497	Iron ion binding; oxidoreductase activity, acting on paired donors, with incorporation or reduction of molecular oxygen; heme binding; gamma-tubulin complex localization; monooxygenase activity
	OG0011385	GO:0005515	Protein binding
	OG0011444	GO:0003860	3-Hydroxyisobutyryl-CoA hydrolase activity, mitochondrial

**Table 3.** Gene Ontology terms significantly enriched in accessory genes.

	GO ID	Term	Genes with term	Accessory genes with term	*P*-value (Fisher test)
Biological process	GO:0031503	Protein-containing complex localization	9	3	0.001
	GO:0006468	Protein phosphorylation	158	9	0.01
	GO:0006355	Regulation of transcription, DNA-templated	287	14	0.01
	GO:0032502	Developmental process	8	2	0.02
Molecular function	GO:0008757	S-adenosylmethionine-dependent methyltransferase activity	33	4	0.003
	GO:0004672	Protein kinase activity	170	9	0.01
	GO:0000981	DNA-binding transcription factor activity, RNA polymerase II-specific	177	9	0.01
	GO:0016289	CoA hydrolase activity	8	2	0.01
	GO:0008270	Zinc ion binding	352	12	0.04
	GO:0003700	DNA-binding transcription factor activity	221	12	0.04

There were a total of 888 secreted orthogroups, which were sub-classified as 223 effectors (149 apoplastic and 74 cytoplasmic), and 665 non-effectors (Supplementary Table 2). The accessory secretome had a significantly higher proportion of effectors (44%) than the core secretome, which only had 24% effectors (the 2 proportion z-test, *P*-value < 0.05) (Table 4). The opposite was true for the proportion of non-effector secreted orthogroups, which was significantly higher in the core secretome (the 2 proportion z-test, *P*-value < 0.05) (Table 4).

**Table 4.** The *C. zeina* accessory secretome contains a significantly higher proportion of effectors than the core.

	Core + accessory*^a^*	Core*^b^*	Accessory*^c^*	Significant difference in proportions (*P*-value)*^d^*
Total orthogroups (secreted)	888	854 (100%)	34 (100%)	
Non-effector orthogroups (secreted)	665	646 (76%)	19 (56%)	0.02*
Effector orthogroups (secreted)	223	208 (24%)	15 (44%)	0.02*
				
Effector orthogroups (secreted)—apoplastic	149	139 (16%)	10 (29%)	0.08
Effector orthogroups (secreted)—cytoplasmic	74	69 (8%)	5 (14%)	0.29

Number of orthogroups in core plus accessory secretome.

Number of orthogroups in core secretome (percentage of orthogroups in core secretome are written in brackets).

Number of orthogroups in accessory secretome (percentage of orthogroups in accessory secretome are written in brackets).

Test for enrichment of orthogroup type in accessory compared to core secretomes. Two-sample test for equality of proportions with continuity correction; asterisk indicates a significant difference (alpha = 0.05).

In addition, we aimed to determine whether accessory genes were associated with genomic regions containing repetitive and mobile elements. We made use of the well-assembled PacBio genome of the reference *C. zeina* isolate CMW25467, which contained the 10,677 core genes and 394 accessory genes. In a comparison of the median distance of the genes to the nearest TE, the accessory genes were significantly closer to a TE than the core genes were (mean 1.7 Kb) (Wilcoxon rank sum test, *P*-value = 0.018) (Supplementary Fig. 2).

### Population differentiation between East and Southern African *C. zeina* isolates

A further goal of this study was to determine the population genetic structure of *C. zeina* isolates from the 5 African countries based on genome-wide SNP data. To this end, we conducted genome-wide SNP calling based on a reference assembly to identify variable sites along the pathogen genomes (Table 1). We hypothesized that differences in environment or farming practices between East and Southern Africa could result in population differentiation and local adaptation. For example, Kenya and Uganda are on the equator, whereas the maize growing region of KwaZulu-Natal province in South Africa is at a latitude of 30° South. Furthermore, maize is grown continuously in East Africa, whereas there is a single summer season in South Africa (Nsibo *et al*. 2021).

Based on the SNP calling results of the 30 African *C. zeina* isolates compared to the reference genome of *C. zeina* CMW25467 (Table 1), we identified a total of 151,074 SNPs (after quality filtering, see Materials and methods). Furthermore, we called 8,226 indels, but these were not used in our downstream analyses.

**Fig. 3.**
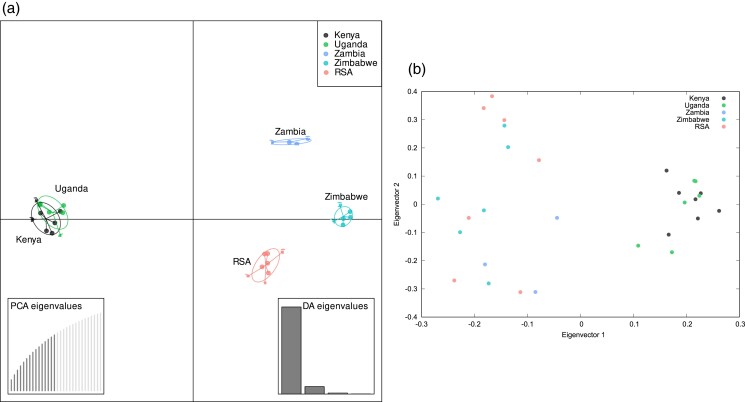
Genome-wide SNP analysis of the 31 *C. zeina* isolates shows differentiation into an East African cluster (Uganda, Kenya) and a Southern African cluster [Zambia, Zimbabwe, South Africa (RSA)]. a) DAPC using 151,074 biallelic SNPs shows the regional differentiation between isolates. Isolates from each country are color-coded: Kenya (black), Uganda (green), RSA (South Africa) (orange), Zambia (blue), and Zimbabwe (cyan). b) PCA plot with stratification correction produced using Eigensoft shows a similar regional differentiation of the isolates as the DAPC analysis. This analysis was based on 2,156 LD-pruned SNPs present in 2 or more isolates. Isolates were color-coded the same as in the DAPC plot.

We employed 3 methods to explore the population structure among the *C. zeina* populations. First, we conducted a DAPC using the complete SNP dataset to identify patterns of population clusters. The analyses revealed a clear separation along the first principal component (x-axis) between isolates from East Africa (Kenya and Uganda), and isolates from Southern Africa (Zambia, Zimbabwe, South Africa) (Fig. 3a). Interestingly, DAPC analysis did not differentiate the isolates from Kenya and Uganda, whereas there was differentiation on the second principal component (y-axis) between Zambia, Zimbabwe, and South Africa (Fig. 3a). We conducted an additional PCA analysis with correction for stratification to explore the population structure of the 31 *C. zeina* isolates. This analysis was carried out with a subset of 2,157 Linkage Disequelibirum (LD)-pruned SNPs [minor allele frequency (MAF) > 6.6%] in the software Eigensoft (Price *et al.* 2006). Again, the East African (Uganda and Kenya) *C. zeina* isolates clustered together and were differentiated from the isolates from the Southern African countries (Fig. 3b, Eigenvector 1 on x-axis).

**Fig. 4.**
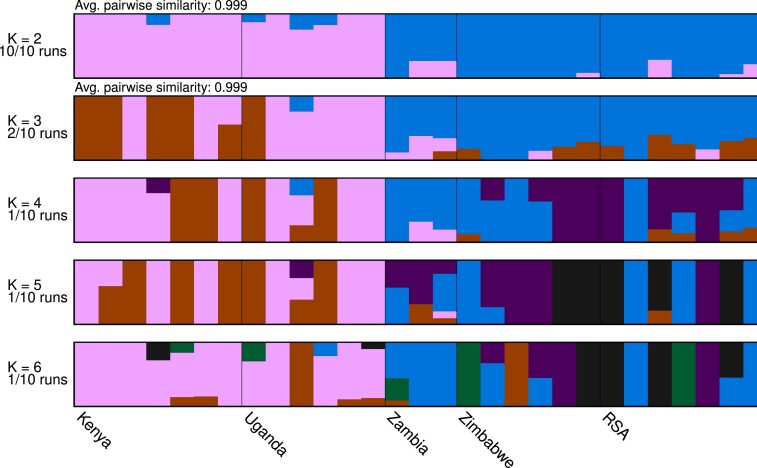
Admixture analyses between the 31 isolates of *C. zeina* converged into 2 subpopulations of inferred ancestry (K = 2) in all 10 runs, namely an East African group (Uganda, Kenya) and a Southern African group (Zambia, Zimbabwe, RSA). The analyses used 2,156 LD-pruned SNPs present in 2 or more isolates. Implementing different clusters of isolates (K = 3 to K = 6) did not provide consistent patterns of inferred ancestry.

Next, we explored the ancestry of the 31 *C. zeina* isolates (including also the reference isolate) using the program ADMIXTURE with the subset of 2,157 LD-pruned SNPs. The analysis provided highest support for 2 groups of shared ancestry (K = 2, Fig. 4) with 1 from East Africa (Kenya and Uganda) and the other from Southern Africa (Zambia, Zimbabwe, South Africa). A clustering model of K = 3 indicated some differentiation between the Zambian isolates and the Zimbabwean and South Africa isolates (Fig. 4) as seen for the DAPC analysis. Higher clustering models (K = 4 to K = 6) did not consistently resolve the populations.

**Fig. 5.**
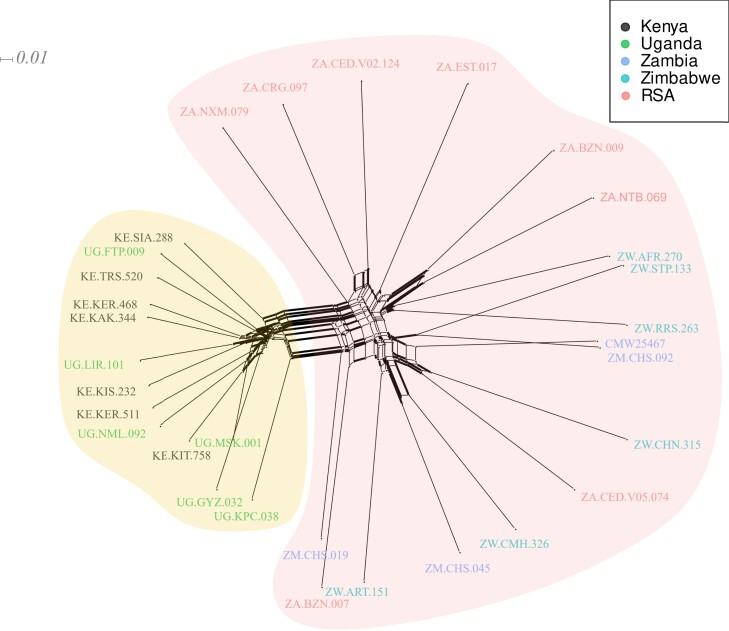
Phylogenetic neighbor-net network analysis separates the 31 *C. zeina* isolates broadly into East African and Southern African clusters. There were 88,023 biallelic SNPs present in 2 or more isolates that were used for the whole-genome comparisons. The widths of the edges represent their confidence values, with the thick edges representing >80% confidence. The scale bar illustrates branch distances. *C. zeina* isolate names are color-coded per country: Kenya (black), Uganda (green), South Africa (red), Zambia (blue), and Zimbabwe (cyan).

Finally, we explored the relationships between the African *C. zeina* isolates with a phylogenetic approach using 88,023 biallelic genome-wide SNPs with MAF > 6.6%. The neighbor-net network based on whole-genome distances showed an overall separation of East African isolates from Southern African isolates (Fig. 5). However, there is a large amount of reticulation evident in the splitstree indicating recurrent and recent recombination and gene flow between isolates in both regions. Furthermore, there was little evidence of country-specific clusters, since there is little differentiation between Kenyan and Uganda isolates, or between Zambian, Zimbabwe, or South African isolates (Fig. 5).

Taken together, the population genomic analyses point to population differentiation between *C. zeina* isolates from East and Southern Africa, but also gene flow between countries of the regions. We speculate that this pattern reflects a recent introduction of 2 founder populations of this pathogen into East and Southern Africa followed by dispersal and recurrent gene flow between countries, and insufficient isolation time for divergence of country-specific lineages. These findings are consistent with a hypothesis that *C. zeina* was introduced to Africa together with maize within the last 500 years (McCann 2007).

## Discussion

### Assembly of the African *C. zeina* pangenome

The accessory genome of the *C. zeina* population in Africa sampled in this study was relatively small compared to some other fungal plant pathogens known to have accessory genomic compartments (Möller and Stukenbrock 2017). Here, based on 31 genomes, we show that the *C. zeina* pangenome has only 7% accessory gene content compared to the 10 to 57% found in other fungal pathogens (Plissonneau *et al.* 2018; McCarthy and Fitzpatrick 2019; Gourlie *et al.* 2022). This was not due to the sample size as, based on the accumulation curve, we showed that adding more African isolates did not add significantly more accessory genes.

Based on previous work done on the microsatellite data of the same African *C. zeina* collection, we hypothesized to find a considerable variation in gene content. This hypothesis was built on the fact that the *C. zeina* population has a high level of genetic diversity and a high level (approximately a third of the genome) of TEs (Nsibo *et al.* 2021; Wingfield *et al.* 2022). We did, however, find evidence that the accessory genes were slightly but significantly closer to these mobile elements in the reference isolate than the core genes, suggesting that TEs play a role in the presence–absence variation of accessory genes. Future work could include other species to allow phylogenetic information to be used to validate whether any of the accessory genes originated through genome rearrangements, as in the pipeline developed by Zabelkin *et al.* (2022).

The low amount of accessory gene content of *C. zeina* in Africa could reflect a genetic bottleneck in the pathogen's evolutionary history in Africa (Badet and Croll 2020). A potential reason could be that only a few genotypes of the pathogen entered Africa when maize was introduced in the past 500 years (McCann 2007). An alternative hypothesis is that *C. zeina* made a host jump from an African grass onto maize after this host was introduced into the continent (Crous *et al.* 2006). However, this is not consistent with the widespread distribution of *C. zeina* globally (Wang *et al.* 1998). Furthermore, a host jump would be expected to occur from a narrow genetic base, which in the timeframe of maize introduction to Africa, would be expected to have a higher level of clonality than we have observed (Nsibo *et al.* 2021). Finally, the lack of large differences in effector gene content between isolates in East and Southern Africa may be due to the distribution of maize germplasm with similar QTLs for GLS resistance between breeding programs (Berger *et al.* 2014).

*C. zeina* is also present as a causal agent of gray leaf spot disease in the center of origin of maize (Mexico), and large-scale maize production on 3 other continents, especially the USA, China, and Brazil (Wang *et al.* 1998, Brunelli *et al.* 2008, Liu and Xu 2013). Therefore, expanding the study to *C. zeina* populations from other continents could be a valuable future research strategy. For example, a larger *C. zeina* pangenome on another continent could indicate that there was a bottleneck after introduction to the African continent. Signatures of selection related to a host shift could then be investigated to potentially resolve the ancestral *C. zeina* population. Bottlenecks lower genetic diversity in general, which would include shared gene presence or absence, but not necessarily overall genome size. For example, a bottleneck occurred in *Z. tritici* after its introduction to Australia, but subsequently, the pathogen underwent a genome expansion and the Australian isolate actually had a larger accessory component compared to the isolates from countries with older populations (Plissonneau *et al.* 2018). A difference in pangenome size would therefore not directly indicate whether the bottleneck was due to an ancestral host shift or invasion into Africa, but phylogenetic approaches including a global collection of isolates could potentially resolve the question of where *C. zeina* originated.

### Accessory genes in *C. zeina* are enriched with effector candidates

In total, we identified 790 accessory genes, several of which could not be assigned a functional relevance. However, for 401 genes we could either classify the genes under a GO term, or we could identify them as genes encoding secreted proteins (34 in total). Although mating type genes cannot be defined as “accessory” in a heterothallic fungus such as *C. zeina*, the GO term “developmental process” was enriched due to presence of either *MAT1-1* (OG0011054) or *MAT1-2* (OG0011238) in each of the 31 isolates (Table 3). This provided an internal validation that our pipeline was able to identify genes present in only some isolates.

The top 2 enriched GO terms in the accessory gene set were protein-containing complex localization and protein phosphorylation. These terms have also been found to be significantly enriched in the accessory genes of other fungal species (McCarthy and Fitzpatrick 2019). Out of the accessory genes annotated with the protein phosphorylation terms, 1 (OG0010814) contained protein domains characteristic of a tyrosine kinase, and another 2 (OG0010691, OG0011136) contained serine/threonine-protein kinase domains. Serine/threonine-protein kinases have been shown to play a role in mating and pathogenicity in other fungal plant pathogens (Zhao *et al.* 2007).

One of the accessory genes (OG0011402) annotated with GO term “protein-containing complex localization” contains protein domains characteristic of a group IV cytochrome P450 (Table 3). This type of protein plays a variety of roles in fungi, including mycotoxin biosynthesis, detoxification, and gibberellin biosynthesis (Črešnar and Petrič 2011). Gaining this gene potentially strengthens a *C. zeina* genotype's ability to compete against other plant pathogens present in the host. This cytochrome P450 was the only enriched accessory gene which was regionally restricted. It was found in only 4 isolates from East Africa and was not present in any Southern African isolates including the reference genome isolate.

Regulation of transcription was also significantly enriched among the accessory genes. There is precedent for accessory transcription factors playing a role in pathogenicity in *Fusarium* (van der Does *et al.* 2016) and *Aspergillus flavus* (Fountain *et al.* 2020). Four (OG0010726, OG0011289, OG0011377, OG0010745) accessory genes annotated with this term contained protein domains characteristic of the same kind of transcription factor, a Zn(2)-C6 fungal-type DNA-binding domain profile. One of them (OG0010745) also contained an aflatoxin biosynthesis regulatory protein signature. Additionally, a Zn(2)-C6 fungal-type transcription factor (OG0000043) was one of the core genes which contained structural SNPs in the coding region. Despite these transcription factors being annotated with similar protein domains, the amino acid sequences were very dissimilar overall between the genes and they were found in different groups of isolates. There was also another enriched accessory gene transcription factor (OG0011345), which contained a different domain for a fungal-specific transcription factor. This is a promising candidate to investigate adaptation in *C. zeina*.

One of the accessory genes with a potential role in local adaptation was a magnesium transporter (OG0000038; Supplementary Table 2). Such a gene has been shown to increase aluminum resistance in *S. cerevisiae* (MacDiarmid and Gardner 1998). Aluminum toxicity is a significant factor in maize fields in East Africa (Lal and Singh 1998; Matonyei *et al.* 2020). Soil acidity (including aluminum toxicity) tends to occur in tropical climates (Von Uexküll and Mutert 1995). The allele in the Eastern African isolates therefore likely reflects adaptation to local abiotic conditions in the Eastern African population.

The accessory component of *C. zeina* contained a greater proportion of predicted effectors than in the core, as has been previously observed in fungal pathogens (Möller and Stukenbrock 2017; Bertazzoni *et al.* 2018). For example, the accessory component was enriched for effector genes in the pangenome of a global collection of 19 *Z. tritici* strains (Badet *et al.* 2020). Accessory effectors are sometimes localized on an accessory chromosome, such as the SIX effectors in *Fusarium oxysporum* (Ayukawa *et al.* 2021) or the *AvrLm11* effector in *L. maculans* (Balesdent *et al.* 2013). Here, in *C. zeina*, the alternate case was observed, where effectors were interspersed in the core chromosomes, such as in *Cochliobolus* (Condon *et al.* 2013). It was previously observed in the CMW25467 PacBio assembly that effectors were not associated with specific genome compartments (Wingfield *et al.* 2022), and the same pattern of genomic locations was observed for the effectors for the other African *C. zeina* isolates sequenced in this study. In ongoing work, knowledge of the *C. zeina* effector candidates should be used to select isolates for pathogenicity trials to investigate which genes are causing a *C. zeina* isolate to be virulent or avirulent on particular maize genotypes.

### Population differentiation between East and Southern African *C. zeina* isolates

Genome-wide SNP marker analysis of the 31 *C. zeina* isolates revealed that there was a level of population differentiation between isolates from East African countries compared to Southern African countries (Figs. 3–5). These analyses included 13 isolates from East Africa and 17 isolates from Southern Africa. However, there was strong evidence for recombination and recurrent gene flow between countries and regions. These results are consistent with a previous study of 964 *C. zeina* isolates from the same countries analyzed with microsatellite markers (Nsibo *et al.* 2021). A level of regional population differentiation between East and Southern Africa was also seen in this study, but considerable evidence for gene flow and migration was seen between countries and regions (Nsibo *et al.* 2021). No clones were included in the 31 isolates used in this study, however, there is considerable evidence for asexual reproduction in the African population with clonal fractions of 7–20% per country for the 964 *C. zeina* isolates (Nsibo *et al.* 2021).

Several hypotheses can be suggested to explain the current population structure of *C. zeina* in Africa. First, the differentiation between East and Southern African populations could be explained by (1) recent but separate introduction of genetically distinct founder populations; or (2) different environmental or maize genotype selection pressures in each region. In support of the first explanation, records indicate that the earliest introductions of maize into West Africa from the Caribbean included whole plant material (McCann 2007). *C. zeina* is not known to be seed-borne, and therefore, introductions would need to be on infected leaf or ear sheath material. Breeding programs, which rely on the introduction of maize germplasm, have generally operated separately in East Africa compared to Southern Africa (Smale *et al.* 2013). This implies that maize material from different sources could have been introduced separately to the East and Southern African regions. In support of this, the population genetics study with microsatellite markers showed that *C. zeina* populations from Kenya and South Africa showed different but high diversity indicating the possibility of 2 founder populations (Nsibo *et al*. 2021).

Farming practices can impact the genetic diversity of plant pathogens. For example, the diversity of rice blast pathogen was greater in fields planted to mixtures of rice genotypes compared to monocultures (Zhu *et al.* 2000). In the maize-*C. zeina* pathosystem, this has also been seen with greater genetic diversity in small-holder farms than large-scale commercial farms in South Africa (Nsibo *et al*. 2019). In the current study, the production practices differ between East and Southern Africa with continuous maize production in East Africa in contrast to a single summer season in South Africa. Furthermore, farmers plant a variety of genotypes, such as open pollinated varieties as well as hybrids in Kenya and Uganda, in contrast to South Africa where hybrids dominate (Berger *et al.* 2020).

Host genotype can influence the genetic diversity of pathogen populations, as was seen in the potato-late blight pathosystem (Stellingwerf *et al.* 2018). Our study showed a slight enrichment of pathogenicity effector genes in the accessory gene content of the African *C. zeina* isolates, although there was not a regional influence. Nevertheless, this points to the effect of host genotype on the *C. zeina* genetic diversity in Africa. It is highly likely that maize germplasm and resistance sources could differ in East and Southern Africa, due to different breeding programs (Smale *et al.* 2013). Currently, breeders rely on quantitative disease resistance to *C. zeina*, and many QTL have been identified in diverse germplasm sources (Berger *et al.* 2014). Maize breeding in East Africa has generally been carried out by National programmes and institutes of the Consultative Group in International Agriculture (CGIAR) such as CIMMYT and IITA, and has made use of tropical maize germplasm with a focus on white maize for human consumption (Worku *et al.* 2020). In contrast, maize production in South Africa and Zimbabwe has been built on breeding with sub-tropical maize germplasm by local companies, national programs, and multinationals (Berger *et al.* 2020). Much of the production in South Africa is yellow maize for animal feed.

Finally, the clustering of *C. zeina* isolates that was determined with a neighbor-net network of *C. zeina* in the 5 African countries shows reticulation indicative of recurrent gene flow and recombination (Fig. 5). Maize production is essentially geographically continuous from East to Southern Africa since it is the staple crop in all sub-Saharan African countries from Ethiopia to South Africa (Smale *et al.* 2013). Spores of *C. zeina* can disseminate from field to field by wind (Ward *et al.* 1999), and due to overlapping seasons, it is feasible for the pathogen to move incrementally from country to country. There are no major mountainous geographic barriers since the Rift Valley runs from North to South. The Zambezi valley between Zambia and Zimbabwe is a West–East barrier, however, it is not devoid of maize production. This may explain why there are shared alleles throughout sub-Saharan Africa. Furthermore, rare events of human mediated dispersal may have occurred, as inferred by the presence of the same microsatellite genotypes in Kenya, Uganda, and a maize disease resistance breeding station (Cedara) in South Africa (Nsibo *et al.* 2021).

Further genetic evidence for recombination in African populations of *C. zeina* is forthcoming from this study and a previous study utilizing microsatellite markers (Nsibo *et al*. 2021). Although the sexual stage of *C. zeina* has not been observed (Crous *et al.* 2006), several lines of evidence support the occurrence of cryptic sex in populations of *C. zeina*. Isolates with both mating types are generally found in all fields surveyed, and mating type ratios are predominantly close to 1:1 in populations at different spatial scales (province or country) in Africa (Muller *et al.* 2016, Nsibo *et al.* 2019, 2021). The clonal fraction of *C. zeina* isolates from small-holder and commercial farms in South Africa were only 11 and 32%, respectively (Nsibo *et al.* 2019).

The signal of recurrent gene flow seen from our genome-wide SNP data from 31 *C. zeina* isolates in the 5 African countries (Fig. 5) may also reflect an intra-specific hybrid swarm, considering that there is evidence for cryptic sex (Nsibo *et al.* 2021). This hypothesis may be difficult to test without knowledge of the 2 or more original parental lineages. This may be forthcoming from sequencing of *C. zeina* populations from other continents. However, 2 factors argue against a hybrid swarm as a complete explanation of population structure observed in this study. First, it is difficult to reconcile how, in the short period of maize cultivation in Africa, that a single intra-specific hybridization event in 1 region and subsequent radiation could result in the observed signal of 2 regional population groups in East and Southern Africa. Second, there is a significant level of clonality in the African population of *C. zeina* (Nsibo *et al.* 2021), invoking considerable asexual reproduction.

In summary, we here provide a comprehensive analysis of genomic variation in an important maize pathogen on the African continent. We find evidence for East and Southern African population structure with some gene flow between countries based on genome-wide polymorphisms. The pangenome of *C. zeina* in Africa moreover comprises several hundred genes exhibiting presence–absence variation. Future studies should aim to unravel the functional relevance of virulence-related candidate genes in this important pathogen.

## Supplementary Material

jkad214_Supplementary_Data

## Data Availability

Illumina sequence reads are available from the Sequence Read Archive (SRA) at NCBI under the BioProject ID PRJNA932176. Scripts have been uploaded to GitHub (https://github.com/twelgemoed/CzeinaPopGen). Supplemental material available at G3 online.
