# Supplementary material for: Population genomic analyses suggest recent dispersal events of the pathogen Cercospora zeina into East and Southern African maize cropping systems
Source: G3 (Bethesda). 2023 Sep 20;13(11):jkad214. doi: 10.1093/g3journal/jkad214 (PMC10627275; doi:10.1093/g3journal/jkad214)

Fig. S1. The 31 selected isolates from the African *Cercospora zeina* collection represent a wide selection of genetically distant genotypes. The UPGMA tree was produced from the SSR genotypes from Table S2 in Nsibo et al. (2021) Fungal Genetics and Biology 149 (103527):1-14. The sampled isolates are labeled and coloured where isolates collected from the same country have the same colour as shown in the legend.

Kenya
Uganda
Zambia
Zimbabwe
RSA

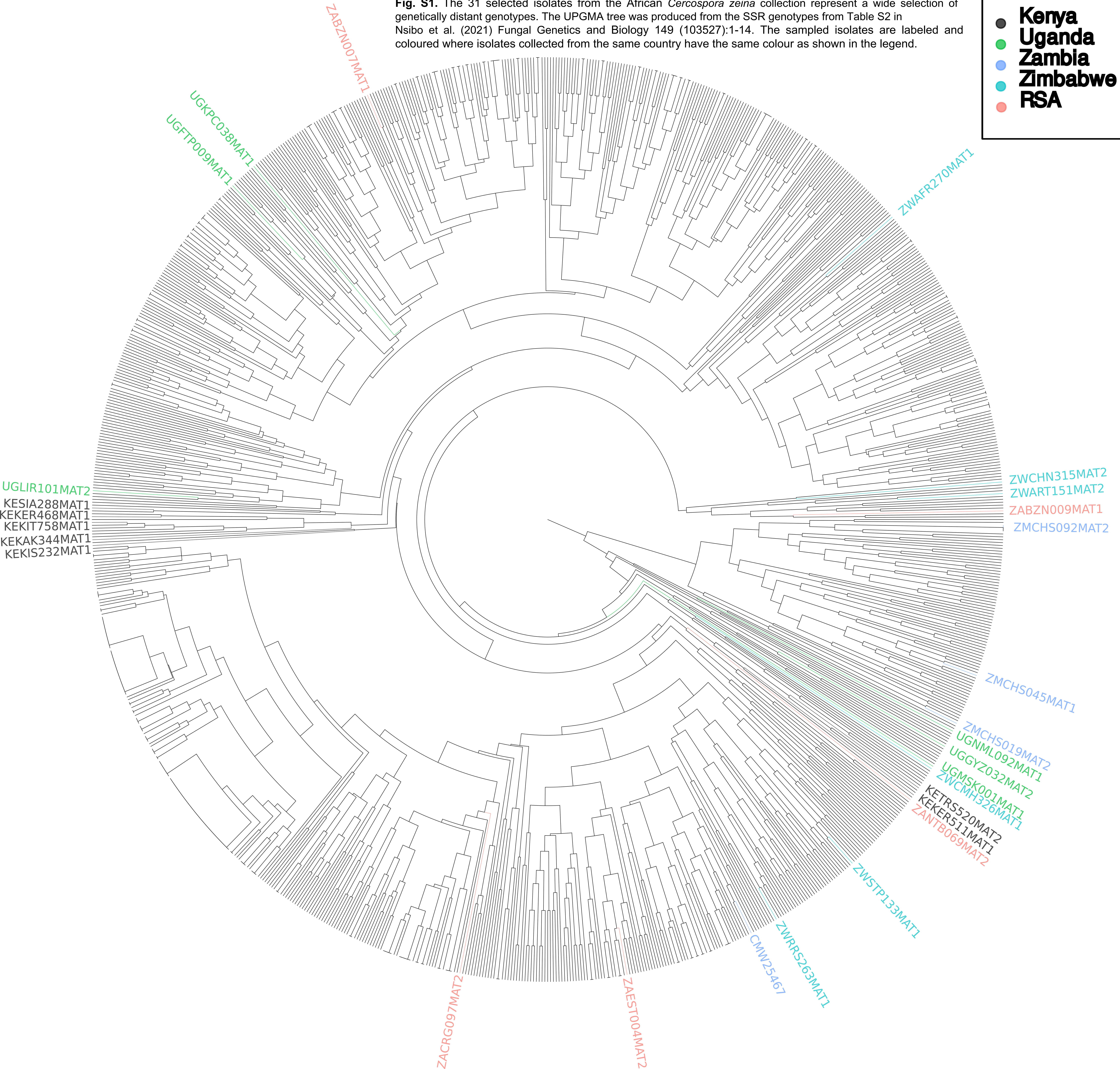

Supplement: jkad214_Supplementary_Data [file jkad214_supplementary_data.zip › Figure_S1_G3-2023-404456.pdf]
