# Supplementary material for: Population genomic analyses suggest recent dispersal events of the pathogen Cercospora zeina into East and Southern African maize cropping systems
Source: G3 (Bethesda). 2023 Sep 20;13(11):jkad214. doi: 10.1093/g3journal/jkad214 (PMC10627275; doi:10.1093/g3journal/jkad214)
Supplement: jkad214_Supplementary_Data [file jkad214_supplementary_data.zip › Figure_S2_G3-2023-404456.pdf]

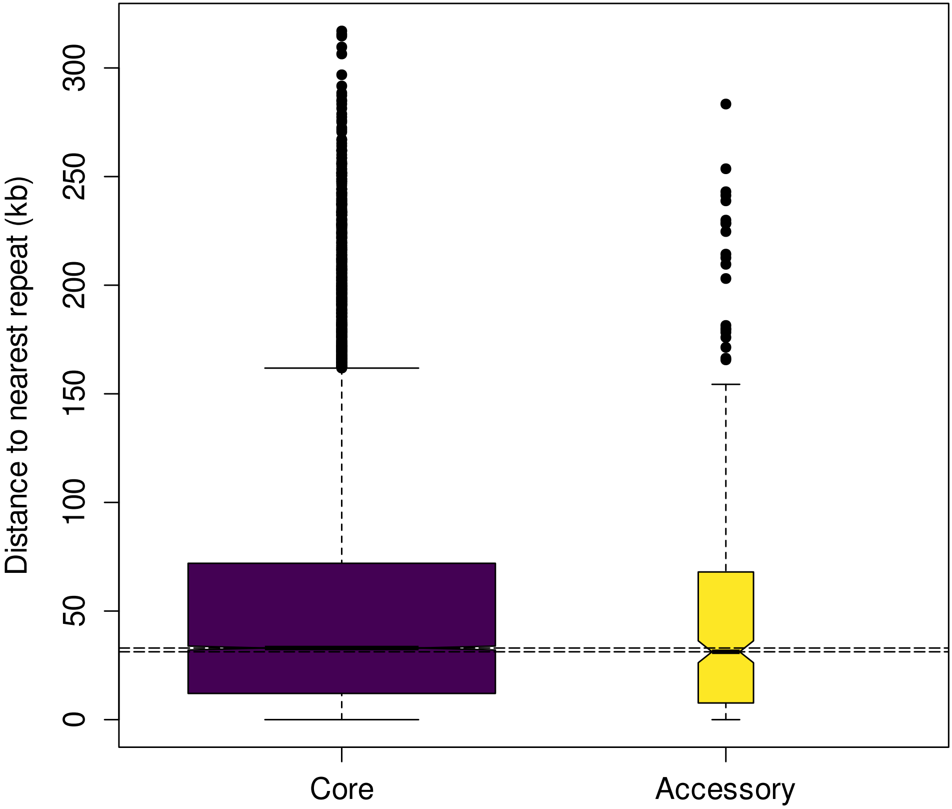


**Figure S2.** The localization of the 10,677 core and 394 accessory genes relative to the transposable repeat elements in the PacBio genome of *Cercospora zeina* CMW25467. The upper and lower horizontal dotted lines indicate the median distances of the Core and Accessory genes to the nearest transposable element, respectively. The difference between the two dotted lines is 1.7 kb.
