# Supplementary material for: Population genomic analyses suggest recent dispersal events of the pathogen Cercospora zeina into East and Southern African maize cropping systems
Source: G3 (Bethesda). 2023 Sep 20;13(11):jkad214. doi: 10.1093/g3journal/jkad214 (PMC10627275; doi:10.1093/g3journal/jkad214)
Supplement: jkad214_Supplementary_Data [file jkad214_supplementary_data.zip › List_of_Supplementary_Files_G3-2023-404456.pdf]

List of Supplementary Files

Population genomic analyses suggest recent dispersal events of the pathogen *Cercospora zeina* into East and Southern African maize cropping systems
Welgemoed et al. 2023

Fig. S1. The 31 selected isolates from the African *Cercospora zeina* collection represent a wide selection of genetically distant genotypes. The UPGMA tree was produced from the SSR genotypes in Table S2 Nsibo *et al.* (2021) Fungal Genetics and Biology 149 (103527):1-14. The sampled isolates are labelled and coloured where isolates collected from the same country have the same colour as shown in the legend.

Fig. S2. The localization of the 10,677 core and 394 accessory genes relative to the transposable repeat elements in the PacBio genome of *Cercospora zeina* CMW25467. The upper and lower horizontal dotted lines indicate the median distances of the Core and Accessory genes to the nearest transposable element, respectively. The difference between the two dotted lines is 1.7 kb.

Table S1. *De novo* genome assemblies and annotations of 30 *Cercospora zeina* isolates from East and Southern Africa compared to the reference isolate CMW25467.

Table S2. Annotation and variation of orthologous gene groups in 31 African *Cercospora zeina* isolates.

Data availability

Illumina sequence reads are available from the Sequence Read Archive (SRA) at NCBI under the BioProject ID PRJNA932176.

Scripts have been uploaded to GitHub (https://github.com/twelgemoed/CzeinaPopGen).
