# Supplementary material for: Population genomic analyses suggest recent dispersal events of the pathogen Cercospora zeina into East and Southern African maize cropping systems
Source: G3 (Bethesda). 2023 Sep 20;13(11):jkad214. doi: 10.1093/g3journal/jkad214 (PMC10627275; doi:10.1093/g3journal/jkad214)
Supplement: jkad214_Supplementary_Data [file jkad214_supplementary_data.zip › Table_S1_G3-2023-404456.pdf]

Table S1: ***De novo*** **genome assemblies and annotations of 30** ***Cercospora zeina*** **isolates from East and Southern Africa compared to the reference isolate CMW25467.**

Isolate	Contigs[b]	N50 (bp)	Genome size (bp)	Predicted proteins	BUSCO completeness[c]	Annotations with GO term	Annotations with BLAST hits[d]	Number of accessory orthogroups
CMW25467[a]	17	3977222	41717156	11071	97%	5817	11015	394
KE.KAK.344	1196	83283	29726730	11161	97%	5824	11157	484
KE.KER.468	1165	79905	29738074	11155	97%	5821	11148	478
KE.KER.511	1160	90244	29892578	11155	97%	5821	11146	478
KE.KIS.232	1156	84933	29555736	11138	97%	5820	11132	461
KE.KIT.758	1263	74006	29597110	11167	97%	5814	11156	490
KE.SIA.288	1236	76307	29647786	11154	97%	5816	11146	477
KE.TRS.520	1140	82856	29310666	11154	97%	5821	11147	477
UG.FTP.009	1146	84844	29636006	11168	97%	5817	11150	491
UG.GYZ.032	1219	80024	29621031	11177	97%	5821	11164	500
UG.KPC.038	1197	83679	29936476	11135	97%	5817	11127	458
UG.LIR.101	1141	83781	29616805	11168	97%	5814	11162	491
UG.MSK.001	1178	82506	29780028	11157	97%	5821	11152	480
UG.NML.092	1159	83865	29567379	11165	97%	5815	11151	488
ZA.BZN.009	1173	80542	29609777	11174	97%	5810	11130	497
ZA.BZN.007	1181	80695	29643828	11142	97%	5831	11164	465
ZA.CED.V02.124	1187	83687	29872172	11156	97%	5826	11150	479
ZA.CED.V05.074	1230	74221	29724590	11174	97%	5822	11164	497
ZA.CRG.097	1163	84526	29575781	11177	97%	5833	11168	500
ZA.EST.017	1193	75450	29154225	11194	97%	5822	11175	517
ZA.NTB.069	1270	75698	29824153	11176	97%	5830	11159	499
ZA.NXM.079	1226	79254	29765393	11144	97%	5819	11132	467
ZM.CHS.019	1200	85458	29616338	11146	97%	5812	11138	469
ZM.CHS.045	1200	82881	29700012	11143	97%	5823	11135	466
ZM.CHS.092	1179	80299	29646602	11144	97%	5816	11142	467
ZW.AFR.270	1222	80926	29854323	11156	97%	5819	11149	479
ZW.ART.151	1135	78658	29284386	11165	97%	5827	11159	488
ZW.CHN.315	1192	85312	29590126	11160	97%	5819	11147	483
ZW.CMH.326	1180	83562	29650334	11164	97%	5826	11158	487
ZW.RRS.263	1199	82352	29683245	11148	97%	5817	11139	471
ZW.STP.133	1188	83739	29664810	11136	97%	5818	11128	459

a Isolate number of *C. zeina* CMW25467 reference genome determined previously with PacBio sequencing (named in Meisel et al. (2009) Eur J Plant Pathol 124 (4):577-583).

b Contigs smaller than 1kbp were filtered out.

c BUSCO analysis used the Dothideomycete conserved protein set

d Diamond blastp against NCBI NR database (E-value < 0.01)
